# Expansion of gene clusters, circular orders, and the shortest Hamiltonian path problem

**DOI:** 10.1007/s00285-017-1197-3

**Published:** 2017-12-19

**Authors:** Sonja J. Prohaska, Sarah J. Berkemer, Fabian Gärtner, Thomas Gatter, Nancy Retzlaff, The Students of the Graphs and Biological Networks Lab 2017, Christian Höner zu Siederdissen, Peter F. Stadler

**Affiliations:** 1 Computational EvoDevo Group, Department of Computer Science and Interdisciplinary Center for Bioinformatics, Universität Leipzig, Härtelstraße 16-18, 04107 Leipzig, Germany; 2 Max Planck Institute for Mathematics in the Sciences, Inselstraße 22, 04103 Leipzig, Germany; 3 Competence Center for Scalable Data Services and Solutions Dresden/Leipzig and Bioinformatics Group, Department of Computer Science, Universität Leipzig, Härtelstraße 16-18, 04107 Leipzig, Germany; 4 Bioinformatics Group, Department of Computer Science and Interdisciplinary Center for Bioinformatics, Universität Leipzig, Härtelstraße 16-18, 04107 Leipzig, Germany; 5 RNomics Group, Fraunhofer Institute for Cell Therapy and Immunology, 04103 Leipzig, Germany; 6 Institute for Theoretical Chemistry, University of Vienna, Währingerstrasse 17, 1090 Wien, Austria; 7 Santa Fe Insitute, 1399 Hyde Park Rd., Santa Fe, NM 87501 USA

**Keywords:** Evolution of gene clusters, Non-homologous recombination, Unequal crossing over, Phylogenetic combinatorics, Kalmanson metrics, Hamiltonian path problems, 92B10, 92D15, 05C45, 05C90, 54E35, 05E45, 62P10

## Abstract

Clusters of paralogous genes such as the famous HOX cluster of developmental transcription factors tend to evolve by stepwise duplication of its members, often involving unequal crossing over. Gene conversion and possibly other mechanisms of concerted evolution further obfuscate the phylogenetic relationships. As a consequence, it is very difficult or even impossible to disentangle the detailed history of gene duplications in gene clusters. In this contribution we show that the expansion of gene clusters by unequal crossing over as proposed by Walter Gehring leads to distinctive patterns of genetic distances, namely a subclass of circular split systems. Furthermore, when the gene cluster was left undisturbed by genome rearrangements, the shortest Hamiltonian paths with respect to genetic distances coincide with the genomic order. This observation can be used to detect ancient genomic rearrangements of gene clusters and to distinguish gene clusters whose evolution was dominated by unequal crossing over within genes from those that expanded through other mechanisms.

**Electronic supplementary material:**

The online version of this article (10.1007/s00285-017-1197-3) contains supplementary material, which is available to authorized users.

## Introduction

The genomes of higher eukaryotes typically contain many families of genes with similar DNA sequence. These usually encode similar proteins and share similar function. Their sequence similarity indicates that they have evolved from a single original ancestor by means of multiple rounds of duplication. Such paralogous genes are often, but by no means always, located at the same genomic locus, where they form a gene cluster. In many cases clustered genes are not tied together functionally and the clusters can disintegrate by genome rearrangement without detrimental effects.

However, some gene clusters are evolutionarily old and have retained a very particular organization of their member genes for hundreds of millions of years. Among the best characterized gene clusters are the globin gene clusters, which encode major players in the transport of oxygen within the bloodstream (Maniatis et al. 1980) and the homeobox Hox gene clusters, which play a crucial role in the early stages of animal development (Garcia-Fernàndez 2005). In vertebrates, the latter show very low levels of repeats and unrelated open reading frames, and the genes in paralogous clusters share the same order and orientation. Experimental work demonstrated that the consolidated arrangement is crucial and constrained due to the necessity of a coordinated regulation orchestrated by enhancer sequences outside the cluster (Hardison et al. 1997; Montavon and Duboule 2013).

**Fig. 1:**
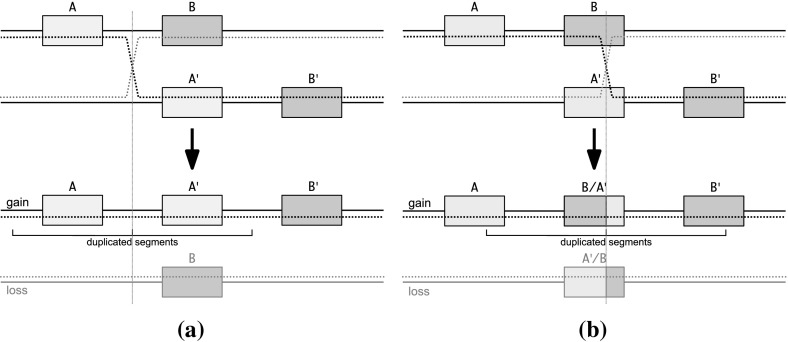
Gene cluster expansion by local gene duplication (**a**), unequal crossing over in Gehring’s model (**b**). During mitosis, when chromatids are paired, unequal crossing leads to a tandem duplication on one chromatid and a deletion on the sister chromatid. The loss of whole genes is considered to be lethal. In Gehring’s model the crossing over occurs within the gene sequences resulting in hybrid genes. Crossing over between intergenic sequences results in duplication of complete genes

The details of the molecular mechanisms and evolutionary forces that govern the expansion of clusters of paralogous genes are by no means completely understood. Walter J. Gehring, a developmental biologist famous for his studies of the Hox gene cluster in *Drosophila melanogaster*, interpreted the fact that the three Hox genes (*abd-B*, *abd-A*, and *Ubx*) appear in a tandem arrangement as evidence for gene duplication by “unequal crossing over”. He proposed that the current Hox cluster expanded from two Hox genes by a series of unequal crossing over events between highly similar but mispaired paralogous genes (Gehring 1998). In this scenario, a new paralog is created as a hybrid of its left and right neighbors as indicated in Fig. 1.

The local gene duplication model constitutes an alternative explanation. Again, unequal crossing over is a molecular mechanism resulting in the duplication. However, in this scenario the crossing over occurs between genes and thus results in the creation of a faithful copy of the complete gene. Diversification, subfunctionalization, or neofunctionalization then drives the subsequent divergence of the paralogous sequences (Ohno 1970; Force et al. 1999).

Gehring noted that terminal genes in a Hox cluster are not subject to changes by crossing over and that the genes in the middle of the cluster are more similar to the consensus sequence than more distal genes. The paralogs in a cluster most similar to a given gene tend to be its neighbors. A recent analysis of the genetic distances, i.e., a suitably transformed measure of sequence similarity (Nei 1972) between Hox genes, furthermore, showed that the shortest Hamiltonian path with respect to the genetic distance follows the genomic order of the cluster (Höner zu Siederdissen et al. 2015). We ask here if and how these observations can be explained by Gehring’s model and the local gene duplication model.

The analysis of the history of a gene family is usually based on the inference of a phylogenetic tree of the paralogous genes in question. However, this is a difficult task and often remains unsuccessful, in particular for the deep branches since several effects conspire to erase the phylogenetic signal. Saturation of the phylogenetic signal limits the power of reconstruction in particular for old events and events separated by relatively short time scales.

Genomic elements that are very similar in sequence and in close proximity, as is the case in clusters of paralogous genes, are particularly prone to gene conversion and other mechanisms of concerted evolution (Carson and Scherer 2009; Noonan et al. 2004). Last but not least, the very process that introduces additional new members may involve unequal crossing over in Gehring’s model thus producing a non-tree-like structure of genetic distances to begin with.

The purpose of this contribution is two-fold. First, we investigate the consequences of Gehring’s model for gene cluster expansion and show that while the resulting genetic distances are not additive trees, they form a special class of Kalmanson (circular decomposable) metrics (Kalmanson 1975), which we term type R metrics. Circular decomposable metrics are intimately related to weakly compatible split systems (Bandelt and Dress 1992) that admit a circular order (Christopher et al. 1996; Chepoi and Fichet 1998). Our interest in circular orderings in this context is far from accidental: There is a large body of literature that not only explores these connections in detail (Farach 1997; Dress et al. 2000; Kleinman et al. 2013); circular decomposable metrics and their associated split systems also form the basis for the most important and widely used practical methods for reconstructing phylogenetic networks: NeighborNet (Bryant et al. 2004, 2007) and Qnet (Grünewald et al. 2007, 2009). Second, we will see that in the absence of extreme selective pressure they have the Robinson property, which ensures that the Hamiltonian path with the shortest genetic distance between genes is co-linear with the genomic order in the gene cluster. We then use this result to distinguish between gene clusters that likely have evolved under Gehring’s model and retained synteny from those that have a different origin or were subject to a rearrangement of their gene order. The contribution is organized as follows: In the following section we survey background material that sets the stage for a detailed analysis of Gehring’s model, which we formalize in Sect. 3 in terms of type R metrics. We then proceed to compare the mathematical model with both simulated and real-life data. A short concluding section, finally, summarizes our findings and points to open questions.

## Trees, metrics, and Hamiltonian paths

In this section we introduce the notation and provide some mathematical background information on the connection between tree metrics and Hamiltonian paths. The material presented in this section is mostly “folklore” and included primarily as an introduction to the more formal development of the following sections. Proofs are included in this section for completeness where we are not aware of any convenient references.

### Gene duplications and genomic gene order

We consider a family *X* of $n=|X|$ paralogous genes whose evolutionary history is given by the tree *T* (with vertex set *V*, leaf set $X\subset V$, and edge set *E*) and strictly positive branch lengths $\ell : E\rightarrow \mathbb {R}^+$. The corresponding genetic distance function $d:X\times X\rightarrow \mathbb {R}_0^+$ is given by

1 $$\begin{aligned} d_{xy} = \sum _{e \in \wp _{xy}} \ell (e) \end{aligned}$$

where $\wp _{xy}$ denotes the unique path connecting *x* and *y* in *T*. We write $d_{\max }=\max _{x,y\in X} d_{xy}$ for the maximal distance between two leaves. It is important here that the genetic distance is additive in the branch lengths and thus proportional to divergence time (Nei 1972). Distance measures counting differences in sequence alignments therefore need to be suitably transformed to additive measures, see e.g. Jukes and Cantor (1969).

Let $\pi :\{1,\ldots ,n\}\rightarrow X$ be a bijection. In other words, $\pi$ defines an ordering of *X* so that $x\prec y$ iff $\pi ^{-1}(x)<\pi ^{-1}(y)$. A special ordering $\widehat{\pi }$ is the arrangement of the genes on the genome.

**Fig. 2:**
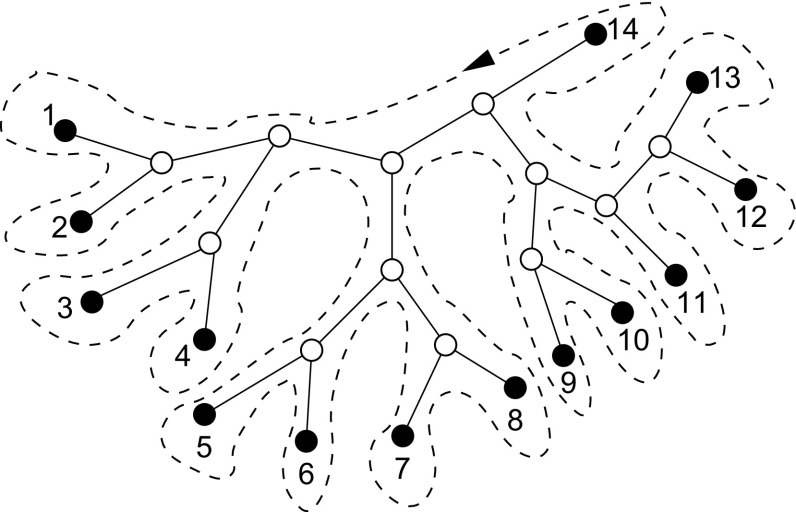
Each planar embedding $\breve{T}$ gives rise to a circular ordering of the vertices by following the “outline” around the tree [see Semple and Steel (2003)]

A *circular* (or *cyclic*) *ordering* (Meggido 1976) is a ternary relation ${\triangleleft {}\,i\,j\,k}$ on a set *X* that satisfies the following five conditions for all $i,j,k\in X$:

1. ${\triangleleft {}\,i\,j\,k}$ implies *i*, *j*, *k* are pairwise distinct. (irreflexive)
2. $${\triangleleft {}\,i\,j\,k}$$ implies $${\triangleleft {}\,k\,i\,j}$$. (cyclic)
3. $${\triangleleft {}\,i\,j\,k}$$ implies $$\lnot {\triangleleft {}\,k\,j\,i}$$. (antisymmetric)
4. ${\triangleleft {}\,i\,j\,k}$ and ${\triangleleft {}\,i\,k\,l}$ implies ${\triangleleft {}\,i\,j\,l}$. (transitive)
5. If *i*, *j*, *k* are pairwise distinct then ${\triangleleft {}\,i\,j\,k}$ or ${\triangleleft {}\,k\,j\,i}$. (total)

A pair of points (*p*, *q*) is adjacent in a total circular order on *V* if there is no $h\in V$ such that ${\triangleleft {}\,p\,h\,q}$. Circular orderings can be linearized by cutting them at any point resulting in a linear order with the cut point as its minimal (or maximal) element (Novák 1984). We will write, by abuse of notation, $i\prec j\prec k$ to mean ${\triangleleft {}\,i\,j\,k}$ together with a suitable linearization, i.e., a cut between *k* and *i*.

It is well known that trees are planar graphs. Let $\breve{T}$ be a fixed planar embedding of *T*. It defines, up to orientation, a unique circular ordering of the leaf set *X*, see e.g. Semple and Steel (2003) for more details. Any linearization of this circular order defines a linear order, which we will refer to as a *T* *-order*, see Fig. 2.

Consider a tree $T=(V,E)$ with leaf-set $X\subset V$ and fix a particular circular order $\pi$ on *X*. Let $E_\pi$ be a set of edges connecting consecutive leaves with respect to $\pi$ and denote by $G_T=(V,E\cup E_{\pi })$ the auxiliary graph with the same vertices as *T* and an edge set extended by $E_\pi$. Thus $G_T$ is a Halin graph (Halin 1971) whenever $\pi$ is *T-order*. A necessary condition for $\pi$ to be a *T-order* therefore is that $G_T$ is a planar graph.

**Fig. 3:**
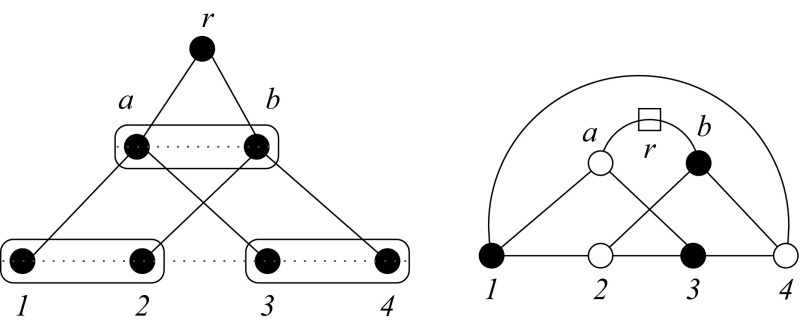
Phylogenetic tree arising from a block duplication of two paralogs. The l.h.s. sketches the phylogenetic tree and the genomic ordering of the leaves. The r.h.s. shows the corresponding graph $G_T$. After contracting the edge between *a* and *r*, we are left with a $K_{3,3}$, hence $G_T$ is not planar. Thus the genomic ordering $\widehat{\pi }$ is not a *T*-ordering

Clearly, if the gene family originated exclusively by tandem duplications, then the genomic order $\widehat{\pi }$ is a *T*-order for the gene phylogeny *T*. On the other hand, if a block containing two or more genes is duplicated as a unit, then $\widehat{\pi }$ and the tree are discordant as shown in Fig. 3. Every duplication scenario in which more than a single gene duplicated at least once must contain this situation as a subgraph, and thus the complete bipartite graph $K_{3,3}$ shown in the right panel of Fig. 3 is a minor. $K_{3,3}$ and the complete graph of five vertices $K_5$ are the two minimal obstructions to planarity, see Makarychev (1997) and the references therein. Thus it follows that $\widehat{\pi }$ is not a *T*-order whenever the evolutionary scenario involves larger block duplications. We remark that gene loss may erase this signature of block duplications. For instance, the loss of node (leaf) 2 or 3 in Fig. 3 leads back to a *T*-order.

### From trees to Hamiltonian paths

For an arbitrary order $\pi$ we define the length function

2 $$\begin{aligned} L(\pi ) = \sum _{i=2}^n d_{\pi (i-1)\pi (i)} \end{aligned}$$

$L(\pi )$ can be interpreted as the length of the Hamiltonian path defined by the ordering $\pi$ in the complete graph with vertex set *X* and edge lengths $d_{xy}$.

#### Theorem 1

Let *d* be the additive tree metric associated with the tree *T* and its non-degenerative length function $\ell$. Then $L(\pi )$ is minimal if and only if (i) $\pi$ is a *T*-order and (ii) $d_{\pi (1)\pi (n)}=d_{\max }$.

#### Proof

We use the abbreviation $\mathcal {L} = \sum _{e\in E} \ell (e)$. $\square$

#### Claim 1

Every order $\pi$ satisfies $L(\pi ) \ge 2\mathcal {L} - d_{\max }$.

Denote by $\omega$ the closed walk $\wp _{\pi (1)\pi (2)}\wp _{\pi (2)\pi (3)} \ldots \wp _{\pi (n-1)\pi (n)}\wp _{\pi (n)\pi (1)}$. Its length is $L(\omega )=d_{\pi (n)\pi (1)}+\sum _{i=2}^n d_{\pi (i-1)\pi (i)}$. Since $\omega$ connects any two leaves, it contains all edges of *T*. Furthermore, since *T* contains no cycle, $\omega$ must leave each subtree that it enters along the same edge. Thus $\omega$ covers any edge at least twice. Hence $L(\omega )\ge 2\mathcal {L}$. Since $\omega$ contains exactly one path too many, and the longest possible path had length $d_{\max }$, the claim follows.

#### Claim 2

If $\pi$ is *T*-order, then $L(\pi ) = 2\mathcal {L} - d_{\pi (1)\pi (n)}$.

By construction $\omega$ associated with a *T*-order is the closed walk defined by the “outline” of the tree, *cf.* Fig. 2. Any such walk covers each edge of *T* exactly twice, once when entering and once when leaving a given subtree. This construction is well known in literature, see e.g. (Moret et al. 2002, Theorem 5). The claim follows directly from $L(\pi )=L(\omega )-d_{\pi (1)\pi (n)}$.

Fix an arbitrary leaf 1 as the root of *T* and a starting and end point of $\omega$ and denote by *n* the last leaf visited for the first time along $\omega$. Furthermore, for every edge *e*, *T*(*e*) denotes the connected component of $T{\setminus }\{e\}$ that does not contain 1.

#### Claim 3

If $\omega$ covers every edge of *T* exactly twice then the leaves contained within every subtree form an interval in $\pi$.

It suffices to note that $\omega$ enters and leaves the subtree *T*(*e*) only through *e*. If the edge is covered exactly twice, all leaves of *T*(*e*), and only the leaves of *T*(*e*) are visited along $\omega$ between the first and the second traversal of *e*.

It follows that, for each edge $e=\{u,v\}$ where $v\in V(T(e))$ and $u\notin V(T(e))$, that is, $T(v) = T(e)$, there is a linear ordering of the children $v_1$, $v_2$, through $v_{d(v)}$ of *v* so that the subtrees $T(v_1)$, $T(v_2), \ldots , T(v_{d(v)})$ are traversed by $\omega$ in this order. Consequently, there is a planar layout of *T* so that the leaves 1 through *n* are arranged in the order of traversal. In other words, if $\omega$ traverses *T* so that every edge is covered exactly twice, then *T* has a planar embedding so that $\omega$ travels along its outline and visits consecutive leaves in the order in which they appear on the outline of the tree.

Hence there is a *T*-ordering following the outline of *T* if and only if the corresponding closed walk covers every edge of *T* exactly twice. Now suppose that $\pi$ is not a *T*-ordering. By closure of the walk, each edge must be covered an even number of times by $\omega$, so that $\omega$ without the return path from $\pi (n)$ to $\pi (1)$ covers at least one edge thrice, thus $L(\pi )> 2\mathcal {L} - d_{\pi (1)\pi (n)}$. $\square$

### Simulating distance matrices for gene duplications

We show here that genetic distance matrices for models of gene duplications can be simulated directly. This has advantages over the more usual approach of simulating sequence evolution. In particular we can, in this manner, separate the stochastic noise that may lead to deviations from additive tree metrics.

#### Lemma 1

Let $d:X\times X\rightarrow \mathbb {R}$ be an additive tree metric on *X* and let $\delta _x\ge 0$ for $x\in X$ be arbitrary. Then $d':X\times X\rightarrow \mathbb {R}$ defined as $d'_{xy}=d_{xy}+\delta _x+\delta _y$ for $x\ne y$ is again an additive tree metric.

#### Proof

A metric *d* is an additive tree metric if and only if every 4-tuple satisfies the “4-point condition” (Buneman 1974; Cunningham 1978; Dobson 1974; Simões-Pereira 1969), which stipulates that any four leaves can be renamed such that

3 $$\begin{aligned} d_{xy}+d_{uv} \le d_{xu}+d_{yv} = d_{xv}+d_{yu} \end{aligned}$$

Using the definition of $d'$ immediately yields

$$\begin{aligned} d'_{xy}+d'_{uv}&= d_{xy}+d_{uv} + \delta _x+\delta _y+\delta _u+\delta _v \\&\le d_{xu}+d_{yv} + \delta _x+\delta _y+\delta _u+\delta _v \\&= d_{xv}+d_{yu} + \delta _x+\delta _y+\delta _u+\delta _v \\&\le d'_{xu}+d'_{yv} = d'_{xv}+d'_{yu} \end{aligned}$$

$$\square $$

Hence we can propagate time by an increment $\Delta t$ simply by adding $\delta _x= r_x\Delta t$ where $r_x$ is the rate of evolution of taxon *x*. A duplication of gene *x* introduces a new gene *z* that, at the time of the duplication event, is identical to *x*. There, it is represented in the distance matrix $\mathbf {D}$ by simply duplicating the row and column *x*, i.e., by setting $d_{zy}=d_{xy}$ for all $y\ne x,z$ and $d_{xz}=0$. The procedure is summarized in Algorithm 1.
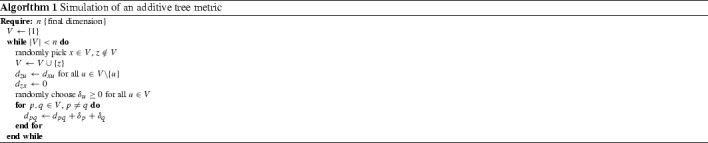


A rate $r_{x'}$ (and possibly a new rate $r_x$) needs to be chosen. Assuming a constant rate of duplication, we set $\Delta t = 1/n$ and choose one of the leaves at random for duplication. Instead of appending the new leaf $x'$ to the end of the matrix, we insert it explicitly before or after *x* so that the order $\pi$ of the rows and columns explicitly encodes the genomic order. Duplicating a larger block of rows and columns can immediately be used to simulate the block duplications of any number of adjacent genes.

#### Lemma 2

Every additive tree metric $d'$ can be constructed by Algorithm 1.

#### Proof

If $d'$ is an additive tree metric, then there is a unique additive tree *T* with edge lengths $\ell :E\rightarrow \mathbb {R}^+_0$ representing $d'$. Suppose for the moment that *T* is binary. Then it has at least one “cherry”, i.e., a pair of leaves separated by only a single interior vertex, say $\{p,q\}$. It is easy to check that every cherry in *T* must satisfy

4 $$\begin{aligned} \min _{x,y\in V{\setminus }\{p,q\}} \left\{ \left( d'_{px}+d'_{qy}\right) - \left( d'_{pq}+d'_{xy}\right) \right\} >0 \end{aligned}$$

If $\{p,q\}$ is a cherry, then the distances in *T* from *p* and *q* to their last common ancestor are $\delta _p=(1/2)\min _{u,v\ne p} \left( d'_{pu}+d'_{pv}-d'_{uv}\right) \ge 0$ and $\delta _q=(1/2)\min _{u,v\ne q} \left( d'_{qu}+d'_{qv}-d'_{uv}\right) \ge 0$, both of which are non-negative as a consequence of the triangle inequality. The reduced distance matrix $\mathbf {D}$ on $V{\setminus }\{q\}$ defined by $d_{xy}=d_{xy}$ for $x,y\notin \{p,q\}$, $d_{xp}=d'_{xp}-\delta _p$ represents *T* with the cherry replaced by its last common ancestor, hence it is again an additive distance matrix.

Repeating this construction we arrive at a single vertex after $|V|-1$ steps. Each step identifies a leaf *p* that is duplicated and the extensions $\delta _p$ and $\delta _q$ of *p* and its copy *q*. Note that we have set $\delta _x=0$ for all $x\in V{\setminus }\{p,q\}$. This reflects that the stepwise elongation of the trees’ branches modeled in Algorithm 1 can be subdivided arbitrarily between duplication events that affect a particular branch. Here we simply choose to add the entire length immediately after each duplication event. Thus the construction in this proof backtraces a particular sequence of duplication events in Algorithm 1.

The case of non-binary trees is easily incorporated by observing that it can be represented as binary tree in which an internal branch length of 0 is also allowed. $\square$

## Type R distance matrices

### Construction and recognition

The model so far corresponds to a mechanism in which unequal crossing over occurs only *between* the genes of interest. We can, however, also model events in which the genes themselves are recombined. Instead of assuming that the newly introduced gene *z* is a true copy of *x*, we now assume that *z* is a recombinant of two adjacent genes *x* and *y*. The product is inserted between *x* and *y*.

Since *z* is composed of two parts, of relatives sizes *a* and $(1-a)$, $0\le a\le 1$, that are identical to *x* and *y*, respectively, we have

5 $$\begin{aligned} \begin{aligned} d_{zu}&= a d_{xu} + (1-a) d_{yu} \quad {\text {for all}}\quad u\ne x,y,z\\ d_{zx}&= (1-a) d_{xy} \\ d_{zy}&= a d_{xy} \\ \end{aligned} \end{aligned}$$

After the duplication event, each gene evolves independently with its own rate, so that the genetic distance between *p* and *q* again grows by $\delta _p+\delta _q$, i.e.,

6 $$\begin{aligned} d'_{pq}=d_{pq} + \delta _p+\delta _q \end{aligned}$$

#### Definition 1

A distance matrix $\mathbf {D}$ is of *type R* if it is constructed by repeated application of Eqs. (5) and (6).

Clearly, every additive tree metric, and thus also every phylogenetic tree resulting from tandem duplications is of type R by virtue of setting $a=0$ (or $a=1$) in every duplication step. In particular, therefore, for $n=3$ every distance matrix is of type R. For $n>3$, however, it is not obvious whether a type R matrix can be recognized efficiently. The evolutionary history therefore must not include e.g. simultaneous duplications for two or more genes (as in the example of Fig. 3), or genome rearrangements. As we argued above, events will not only violate the type R condition but will in general also interfere with the circular decomposability of the split system.

In order to characterize type R distances, we start by observing

7 $$\begin{aligned} d'_{xz}+d'_{yz}-d'_{xy} = d_{xz}+d_{yz}-d_{xy} + \delta _x+\delta _z+\delta _y+\delta _z-\delta _x-\delta _y = 2 \delta _z \end{aligned}$$

since $$d_{xz}+d_{yz}=(1-a)d_{xy}+a d_{xy}=d_{xy}$$.

For $n\ge 4$, consider the following expression for $u\notin \{x,y,z\}$.

8 $$\begin{aligned} \begin{aligned} d'_{uz}- a d'_{ux}-(1-a) d'_{uy}&= \underbrace{d_{uz}-a d_{ux}-(1-a)d_{uy}}_{=0} \\&\quad + \delta _u+\delta _z-a\delta _u-a\delta _x -\delta _u + a\delta _u -\delta _y+a\delta _y \\&= \delta _z-a\delta _x-(1-a)\delta _y := f(a) \end{aligned} \end{aligned}$$

The key observation is that this expression is independent of *u*. Thus, for $n\ge 5$, there are distinct leaves *u*, *v* distinct from $\{x,y,z\}$ so that $d'_{uz}- a d'_{ux}-(1-a) d'_{uy} = f(a) = d'_{vz}- a d'_{vx}-(1-a) d'_{vy}$, which can be rearranged as $d'_{uz}-d'_{uy} -a d'_{ux} + a d'_{uy} = d'_{vz}-d'_{vy} -a d'_{vx} + a d'_{vy}$ and hence, after a short calculation,

9 $$\begin{aligned} a = \frac{ \left( d'_{uz}+d'_{vy}\right) -\left( d'_{vz}+d'_{uy}\right) }{ \left( d'_{ux}+d'_{vy}\right) -\left( d'_{vx}+d'_{uy}\right) } \end{aligned}$$

Note that this equation must be satisfied for all $u,v\notin \{x,y,z\}$, hence it restricts the space of type R distance matrices to a submanifold for all $n>5$.

Once *a* has been computed, *f*(*a*) can also be computed explicitly. Now consider the following system of equations

10 $$\begin{aligned} \begin{aligned} - a\delta _x - (1-a)\delta _y&= f(a) -\delta _z \\ (1-a) d_{xy} + \delta _x&= d'_{xz} -\delta _z \\ a d_{xy} + \delta _y&= d'_{yz} -\delta _z \\ \end{aligned} \end{aligned}$$

The first line uses the definition of *f*(*a*) above, the second and third line are rearrangements of $d'_{xz}=(1-a)d_{xy}+\delta _x+\delta _z$ and $d'_{yz}= a d_{xy}+\delta _y+\delta _z$, resp. multiplying the second and third line by *a* and $(1-a)$ and adding up the three equations yields $2a(1-a) d_{xy} = f(a) - 2\delta _z + a d'_{xz} + (1-a) d'_{yz}$. We can now compute $d_{xy}$ from

11 $$\begin{aligned} 2a(1-a) d_{xy} = \left( d'_{uz}- a d'_{ux}-(1-a) d'_{uy} \right) - 2\delta _z + a d'_{xz} + (1-a) d'_{yz} \end{aligned}$$

Finally, $\delta _x$ and $\delta _y$ are obtained from

12 $$\begin{aligned} \begin{aligned} \delta _x&= d'_{xz}-(1-a)d_{xy}-\delta _z \\ \delta _y&= d'_{yz}-ad_{xy}-\delta _z \end{aligned} \end{aligned}$$

In summary, therefore, we can obtain, for $n\ge 5$, complete information on the relative arrangement of the parents *x* and *y* and their recombinant offspring *z*. If $a=0$ or $a=1$ in Eq. (9) then *z* is a copy of *x* or *y*, resp. In this case we cannot determine $d_{xy}$ from Eq. (11) since $2a(1-a)=0$. By construction, however, we can just remove *z* from the matrix to obtain the ancestral state.

It remains to determine the values of $\delta _u$ for $u\notin \{x,y,z\}$. This turns out to be not so trivial, since $\delta _u$ is, in contrast to $\delta _x$, $\delta _y$, and $\delta _z$, not uniquely determined by the last unequal crossing over in Gehring’s model event.

**Fig. 4:**
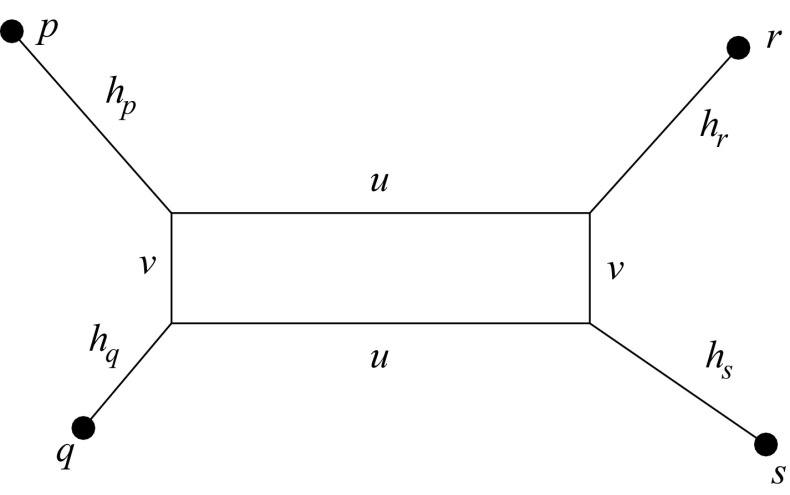
Representation of a metric *d* on 4 points $\{p,q,r,s\}$. Each distance is the sum length of a shortest path in this graph. For instance $d_{pq}=h_p+v+h_q$, $d_{pr}=h_p+u+h_r$, $d_{ps}=h_p+u+v+h_s$

To see this more clearly, let us first consider the case $n=4$. It is well known that every metric on four points can be represented as a “box graph” as shown in Fig. 4. The box dimensions can be computed from $2u=(d_{ps}+d_{qr})-(d_{pq}+d_{rs})$ and $2(u-v)=(d_{rp}+d_{qs})-(d_{pq}+d_{rs})$. The key ingredients thus are the three different pairs of distances emphasized by parentheses. For more details see Nieselt-Struwe (1997). Now let us start from an arbitrary distance matrix $\mathbf {D}$ on $\{x,y,u\}$ and construct *z* as a recombinant. In the following, we will use abbreviations for the three pairs of distance sums, thus

13 $$\begin{aligned} A = d'_{xz}+d'_{uy} \qquad B = d'_{yz}+d'_{ux} \qquad C = d'_{uz}+d'_{xy}. \end{aligned}$$

Using the definitions of $d_{xz}$, $d_{yz}$, and $d_{uz}$ we can compute

14 $$\begin{aligned} \begin{aligned} C-A&= a \left( d_{xy}+d_{xu}-d_{uy}\right) \ge 0 \\ C-B&= (1-a) \left( d_{xy}+d_{yu}-d_{ux}\right) \ge 0 \\ \end{aligned} \end{aligned}$$

using again the triangle inequality. The terms $C-A$ and $C-B$ correspond to twice the sides of the box in the quadruple graph, shown in Fig. 4; note that they are independent of $\delta _x$, $\delta _y$, $\delta _z$, and $\delta _u$. We obtain a tree whenever the box degenerates to a line, i.e., if $a=0$ or $a=1$.

In the general case, the length $h_p$ of the edge incident with leaf *u* becomes $h_p = \delta _p + (1/2)\min _{v,w} (d_{pq}+d_{pr}-d_{qr})\ge 0$, where the minimum runs over all $q\ne r\in V$ different from 0, since we have a box as in Fig. 4 for every quadruple of leaves. It follows that the contribution $\delta _p\ge 0$ that measures that divergence of sequences between duplication events cannot be determined. Intuitively, this comes from the fact that distances are modified by contributions $\delta _u+\delta _v$ deriving from the independent evolution of two leaves. This terms is added to $d_{uv}$ after every duplication event. This contribution cannot be divided unambiguously between the individual steps in complete analogy to the situation for additive tree metrics in the previous section.
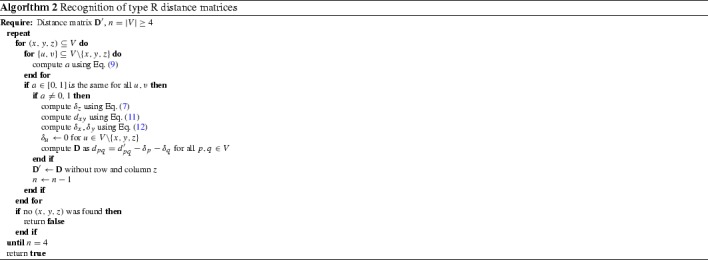


Hence we can set $\delta _u=0$ for every $u\notin \{x,y,z\}$ and assume the entire length of $h_u$ stems from previous events. This yields the recursive Algorithm 2 for recognizing type R distance matrices. It requires *O*(|*V*|) decomposition steps, each of which needs in the worst case $O(|V|^5)$ computations to identify the triple (*x*, *y*, *z*) corresponding to the last duplication event. Note that it suffices to consider $x<y$. If $a=0$ or $a=1$, then *z* was obtained as a faithful copy of *x* or *y*, resp., and hence it can just be dropped. If a candidate triple $\{x,y,z\}$ is found, the previous distance matrix $\mathbf {D'}$ is computed in quadratic time. Thus Algorithm 2 runs in $O(|V|^6)$ time.

For $|V|=4$ the remaining distance matrix is represented by a unique box as in Fig. 4, which implies a unique circular order of the remaining four nodes, say *u*, *x*, *y*, *z*. The fourth node therefore must be the result of unequal crossing over of two nodes that are placed at diagonally opposite corners of the box. Therefore (*u*, *y* : *x*), (*x*, *z* : *y*), (*y*, *u* : *z*), and (*z*, *x* : *u*) are equivalent.

### Linear type R matrices

#### Definition 2

A type R distance matrix is called *linear* (with order $\pi$) if, starting from $V=\{x,y\}$, in each vertex addition step the two parents *x* and *y* are adjacent and their offspring *z* is placed between *x* and *y*.

Algorithm 2 identifies triples (*x*, *y* : *z*) so that *z* was obtained as a recombinant of *x* and *y*, i.e., that *z* is located between *x* and *y* together with a possible temporal order of these events. It is difficult in general to determine whether a linear order exists that is compatible with an arbitrary collection of betweenness triples: the so-called Betweenness Sorting Problem is NP complete (Opatrny 1979; Chor and Sudan 1998). Here, however, we have much more information. We call a type R matrix generic if for every *z* both parents are uniquely defined. We say that (*u*, *v* : *w*) is a successor of (*x*, *y* : *z*) if $\{u,v\}=\{x,z\}$ or $\{u,v\}=\{y,z\}$. A triple without a successor is a leaf triple.

With a leaf triple (*x*, *y* : *z*) we can associate the path $p_{xy}:= x-z-y$. If a triple (*x*, *y* : *z*) has only one successor, say $(x,z:u_1)$, we set $p_{xy}=p_{xz}(z-y)$. If it has two successors, these are of the form $(x,z:u_1)$ and $(z,y:u_2)$, and we set $p_{xy}=p_{xz}p_{zy}$. This is, the paths corresponding to the two “intervals” $x-z$ and $z-y$ are joined at the common vertex *z*. By construction of type R matrices, each triple has at most one predecessor, hence the path $p_{xy}$ is uniquely and completely defined for every triple. A triple (*x*, *y* : *z*) has no predecessor only if *x* and *y* are two of the three ancestral nodes. There are at most two such triples by construction of linear type R matrices, which necessarily have one node in common. The paths are joined at this common node. The type R matrix is linear if the final concatenation result is a single path, in which each node appears exactly once. By construction, *z* is located between *x* and *y* for all triples (*x*, *y* : *z*), i.e., the final path encodes the desired linear order of the nodes.

Representing the paths $p_{xy}$ as lists, joining at their end points can be performed in constant time. Any triple (*x*, *y* : *z*) can be a left or right successor to another triple on (*x*, *y*), accept a left successor on (*x*, *z*), or accept a right successor on (*z*, *y*). For each triple, joining to already processed triples and/or generating references for later triples can be achieved in *O*(1) utilizing the tuple connectors of the triples themselves as keys in associative arrays (one per connection type), e.g. using a quadratic array or (sparse) hash-maps. The successor/predecessor relation between the *O*(*n*) triples can therefore be established in linear time if the triples that account for duplications are already known. Thus, linearity of a type R matrix can be checked in linear time (see Algorithm 3 in the Appendix).

**Fig. 5:**
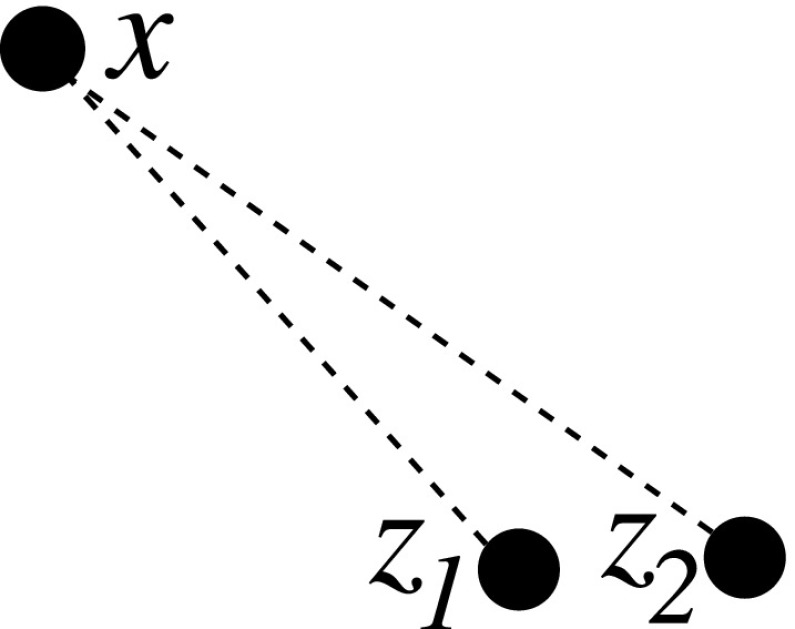
Representation of a successor–predecessor tree after two duplications of the same gene *x*: $(x:z_1)$ and $(x:z_2)$. As the time order of duplications to $z_1$ and $z_2$ are unknown, so is their relation in the genome. Both $x-z_1-z_2$ and $x-z_2-z_1$ are proper solutions

This algorithm can also be extended to the non-generic case. Instances with $a=0$ or $a=1$ duplications result in (*x* : *z*) relations with unknown second flanking gene, which can cause several problems. While the algorithm above can always find one linear configuration, this is no longer unique in the non-generic case. Any pair obtained as “clones” from the same parent have no defined order among themselves, unless a later triple with $0<a<1$ can resolve it (see Fig. 5). Hence, the predecessor–successor relationship is no longer binary, but rather any gene might relate to an unlimited number of perfect copies. This requires careful indexing on individual genes, as listing gene tuples would create exponential growth of open references.

Let us now turn to the connection of type R matrices and circular orders.

#### Definition 3

A distance matrix $\mathbf {D} = (d_{ij})$ satisfies the *Kalmanson condition* if there is a circular order $\triangleleft$ of the points so that the inequality

15 $$\begin{aligned} \max \{ (d_{ij}+d_{kl}), (d_{il}+d_{jk}) \} \le d_{ik}+d_{jl} \end{aligned}$$

for every four points so that $i\prec j\prec k\prec l$.

If $(d_{ij})$ satisfies Eq. (15) then the corresponding Travelling Salesman Problem (TSP) is solved by the unit permutations, i.e., $\pi =(1,2,3,\ldots ,n)$ (Kalmanson 1975). Equivalently, if $\triangleleft$ is a circular ordering of the taxa set *V* and $\pi$ the permutation of *V* associated with an arbitrary linearization of $\triangleleft$, then $(d_{ij})$ is Kalmanson iff

16 $$\begin{aligned} \max \{ (d_{\pi (i)\pi (j)}+d_{\pi (k)\pi (l)}), (d_{\pi (i)\pi (l)}+d_{\pi (j)\pi (k)}) \} \le d_{\pi (i)\pi (k)}+d_{\pi (j)\pi (l)} \end{aligned}$$

for $i<j<k<l$. In this case $L(\pi )$ in Eq. (2) is a shortest Hamiltonian cycle for $(d_{ij})$.

With each circular ordering $\triangleleft$ we can associate a set $\mathcal {S}^{\triangleleft }$ of splits, i.e., non-trivial bipartitions of the set *X* of taxa. $\{A,X{\setminus } A\}\in \mathcal {S}^{\triangleleft }$ if and only if (i) $A\ne \emptyset$, (ii) $A\ne X$, (iii) there is $i,j\in A$ and $k,l\in X{\setminus } A$ so that (a) for all $p\in A$ and $q\in X{\setminus } A$ holds ${\triangleleft {}\,i\,p\,j}$ and ${\triangleleft {}\,k\,q\,l}$ and (b) ${\triangleleft {}\,i\,j\,k}$ and ${\triangleleft {}\,k\,l\,i}$. We write

17 $$\begin{aligned} S_{ij}:=\left\{ \{\pi (i+1),\pi (i+2),\ldots , \pi (j)\}, \{\pi (j+1),\pi (j+2),\ldots , \pi (i)\} \right\} \end{aligned}$$

with *i*, *j* taken $\mod |X|$ for the splits of $\mathcal {S}^{\triangleleft }$, where $\pi$ is again an arbitrary linearization of $\triangleleft$. A metric is called *circular decomposable* (Bandelt and Dress 1992) if there is a circular ordering $\triangleleft$ (with a corresponding permutation $\pi$), and $\alpha _{ij}\ge 0$, $i\ne j$ so that

18 $$\begin{aligned} d_{xy} = \sum _{i<j} \alpha _{ij} \delta _{S_{ij}}(x,y), \end{aligned}$$

where the split pseudometric $\delta _{S_{ij}}$ is defined as $\delta _{S_{ij}}(x,y)=1$ if the split $S_{ij}$ separates *x* and *y*, and $\delta _{S_{ij}}(x,y)=0$ otherwise. Such expressions are known as “Crofton formulas” (Chepoi and Fichet 1998). The *isolation indices* of the splits $S_{ij}$ can be computed as

19 $$\begin{aligned} \alpha _{ij} = \alpha (S_{ij}) = \frac{1}{2}\left( d_{\pi (i)\pi (j)}+d_{\pi (i+1)\pi (j+1)} - d_{\pi (i)\pi (j+1)} - d_{\pi (i+1)\pi (j)}\right) \end{aligned}$$

It is shown by Christopher et al. (1996) and Chepoi and Fichet (1998) that a metric satisfies the Kalmanson condition if and only if it is circular decomposable. These can be represented as so-called split graphs and computed efficiently using the NeighborNet algorithm (Bryant et al. 2004, 2007).

As shown in (Levy and Pachter 2011, Theorem 37) the solution of the TSP on a *generic* circular decomposable metric is unique. Thus, one can use the TSP solutions of $(d_{xy})$ directly for finding circular orderings to be used in NeighborNet (Korostensky and Gonnet 2000; Bryant et al. 2004, 2007). Note that this is not true for special case of additive tree metrics.

#### Theorem 2

Every linear type R distance matrix satisfies the Kalmanson condition.

#### Proof

We only need to show that the distance matrix on $X\cup \{z\}$ is Kalmanson provided the distance matrix on *X* is Kalmanson. Suppose *z* is the recombinant of *j* and $j'$. In the general case we have $i\prec j \prec z \prec j' \prec k \prec l$, since by circularity of the ordering it does not matter whether we duplicate *i*, *j*, *k*, or *l*. In addition to the general case we have to consider the special cases with $i=j$ and/or $j'=k$. The proof repeatedly makes use of the simple observation that $\max (a+p,b+q) \le \max (a,b)+\max (p,q)$.

We assume that the Kalmanson inequalities hold for all quadruples in *X* with an appropriate circular order. For the general case we have, by substituting the definition of the distances involving the recombinant vertex *z*,

$$\begin{aligned} \begin{aligned}&\max \{ d_{iz}+d_{kl}, d_{il}+d_{zk} \} \\&\quad =\max \{ a(d_{ij}+d_{kl}) + (1-a)(d_{ij'}+d_{kl}), a(d_{il}+d_{jk}) + (1-a)(d_{il} +d_{j'j}) \} \\&\quad \le a\max \{ d_{ij}+d_{kl}, d_{il}+d_{jk} \} + (1-a)\max \{ d_{ij'}+d_{kl}, d_{il}+d_{j'k}\} \\&\quad \le a(d_{ik}+d_{jl}) + (1-a)(d_{ik}+d_{j'l}) = d_{ik} + a d_{jl} + (1-a) d_{j'l}\\&\quad = d_{ik}+d_{zl}. \end{aligned} \end{aligned}$$

In the fourth line we use that the Kalmanson inequality holds for $i\prec j\prec k\prec l$ and $i\prec j'\prec k\prec l$ by assumption, the last line used the definition of $d_{zl}$. Analogous computations for the three special cases (omitting the analog of the second and third line above) yield:

$$\begin{aligned} \begin{aligned} \max&\left\{ d_{jz}+d_{kl}, d_{jl}+d_{zk} \right\} \\&\le a\max \left\{ d_{kl}, d_{jl}+d_{jk} \right\} + (1-a)\max \left\{ d_{jj'}+d_{kl}, d_{jl}+d_{j'k} \right\} \\&\le a(d_{jl}+d_{jk}) + (1-a)(d_{jk}+d_{j'l}) = d_{jk}+d_{zl} ;\\ \max&\left\{ d_{iz}+d_{j'l}, d_{il}+d_{zj'} \right\} \\&\le a\max \left\{ d_{ij}+d_{j'l}, d_{il}+d_{jj'}\right\} + (1-a)\max \left\{ d_{ij'}+d_{j'l}, d_{il} \right\} \\&\le a(d_{ij'}+d_{jl}) + (1-a)(d_{ij'}+d_{j'l}) = d_{ij'}+d_{zl} ;\\ \max&\left\{ d_{ij}+d_{zj'}, d_{ij'}+d_{jz} \right\} \\&\le a\max \left\{ d_{ij}+d_{jj'}, d_{ij'} \right\} + (1-a)\max \left\{ d_{ij}, d_{ij'}+d_{jj'} \right\} \\&= a(d_{ij}+d_{jj'}) + (1-a)(d_{ij'}+d_{jj'}) = d_{jj'}+d_{iz}.\\ \end{aligned} \end{aligned}$$

We conclude that all quadruples involving *z* satisfy the Kalmanson inequality provided the distances $(d_{ij})$ form a Kalmanson metric on *V*: we have used the Kalmanson conditions for $i\prec j\prec k\prec l$ as well as the triangle inequality in our proof. As the distances that do not involve the new offspring *z* remain unchanged by the construction principle of type R matrices, we conclude that the distances $(d_{ij})$ on $X\cup \{z\}$ also satisfy the Kalmanson inequalities. $\square$

### Robinsonian distances and Hamiltonian paths

The basic idea of converting a TSP into a shortest Hamiltonian path problem is folklore. One simply adds a dummy node 0 between 1 and *n* with $d_{0\pi (i)}=c$ large enough. Then a shortest Hamiltonian path will use 0 as an endpoint to avoid using 2*c* in the solution. The resulting expanded distance matrix $(d_{ij})$ on $V\cup \{0\}$ is circular decomposable if and only if the Kalmanson conditions also hold for quadruples involving the dummy node, i.e., if and only if

20 $$\begin{aligned} \max \{ d_{0i}+d_{jk}, d_{0k}+d_{ij} \} \le d_{0j}+d_{ik} \end{aligned}$$

holds for all $0\prec i\prec j\prec k$. Since $d_{0i}=c$ this simplifies to the condition

21 $$\begin{aligned} \max \{ d_{ij}, d_{jk} \}\le d_{ik} \qquad {\text {for all}}\quad i<j<k. \end{aligned}$$

A dissimilarity *d* is called Robinsonian if there is a permutation $\pi$ so that

22 $$\begin{aligned} \max \{ d_{\pi (i)\pi (j)}, d_{\pi (j)\pi (k)} \}\le d_{\pi (i)\pi (k)} \qquad {\text {for all}}\quad i<j<k. \end{aligned}$$

The so-called serialization problem (Robinson 1951; Liiv 2010) of linearly ordering objects is solved by the order $\pi$ for Robinsonian dissimilarities. This result appears to be folklore, we have not found a simple direct proof.

#### Lemma 3

If *d* is Robinsonian, then $\pi$ is a shortest Hamiltonian path.

#### Proof

W.l.o.g. we assume $\pi =\iota =(1,2,\ldots ,n)$. Consider an arbitrary permutation $\xi$. Then there is a bijection $\varphi$ between the adjacencies $[\xi (i),\xi (i+1)]$ with respect to $\xi$ and the adjacencies $[p,p+1]$ with respect to $\iota$ so that $\xi (i)\le p<p+1\le \xi (i+1)$. To see this we argue by induction. For $n=2$ the statement is trivial. In general $\xi$ is either (1) the extension of a permutation $\xi '$ on $\{1,2,\ldots ,n-1\}$ by one of the adjacencies [1, *n*] or $[n-1,n]$, or (2) $\xi$ is obtained by inserting *n* into the adjacency $[\xi '(k),\xi '(k+1)] = [u,v]$ with $u=\min (\xi '(k),\xi '(k+1))$ and $v=\max (\xi '(k),\xi '(k+1))$ In case (1) $\varphi$ is the extension of $\varphi '$ by $[1,n]\mapsto [n-1,n]$ or $[n-1,n]\mapsto [n-1,n]$. In case (2) we obtain $\varphi$ from $\varphi '$ by replacing $[u,v]\mapsto [p,p+1]$ with $[u,n]\mapsto [p,p+1]$ and adding $[v,n]\mapsto [n-1,n]$. The Robinson condition (21) implies $d_{\xi (i),\xi (i+1)}\ge d_{p,p+1}$ for $\varphi ([\xi (i),\xi (i+1)])=[p,p+1]$ and hence $L(\xi )\ge L(\iota )$, i.e., $\iota$ is a shortest Hamiltonian path. $\square$

The Robinson property also plays an important role in cluster analysis, where it characterizes certain generalizations of hierarchies (Diday 1986; Kleinman et al. 2013; Préa and Fortin 2014). So-called quadripolar Robinson dissimilarities that also satisfy the Kalmanson condition are studied in some detail by Critchley (1994).

#### Lemma 4

Suppose $(d_{ij})$ satisfies Eq. (21) on *V*. Then the distance matrix on $V\cup \{z\}$ obtained by inserting the recombinant node *z* between adjacent parents $j'$ and $j''$ also satisfies the Robinson condition Eq. (21).

#### Proof

Suppose $j=z$ is the new node derived from parents $j'\prec z\prec j''$. Then for $i<j'$ and $k>j''$ we have $d_{iz}= a d_{ij'}+ (1-a) d_{ij''}$ and $d_{zk}= a d_{kj'}+(1-a) d_{j''k}$. Thus $\max \{d_{iz},d_{zk}\} \le a \max _{j'} (d_{ij'}+d_{j'k}) + (1-a) \max _{j''}(d_{ij''}+d_{j''k}) \le d_{ik}$. The special case $i=j'$, $k>j''$ yields: $d_{j'z}=(1-a)d_{j'j''}$ and thus $\max \{ (1-a)d_{j'j''}, a d_{j'k}+(1-a)d_{j'',k}\} \le a d_{j'k}+(1-a)\max \{d_{j'j''},d_{j'',k}\} \le a d_{j'k}+(1-a) d_{j'k} = d_{j'k}$. An analogous computation works for $i<j'$ and $j''=k$. Finally, for $i=j'$ and $k=j''$ we have, by construction $d_{j'z}=(1-a) d_{j'j''}\le d_{j'j''}$ and $d_{zj''} = a d_{j'j''} \le d_{j'j''}$. $\square$

It is important to note that the choice of $\delta _k$ can destroy the inequality: From $\max \{ d_{ij},d_{jk}\} \le d_{ik}$ we cannot conclude that $\{ d_{ij}+\delta _i+\delta _j, d_{jk}+\delta _j+\delta _k \} \le d_{ik}+\delta _i+\delta _k \}$. Hence, very uneven evolution rates or a mechanism that makes the “middle” genes in a gene cluster evolve much faster can destroy the betweenness conditions. The Robinson condition should be satisfied at least in very good approximation if the evolution rates of the offspring are not too different. Gene conversion, which effectively reduces distances, should make it even easier to satisfy Eq. (21).

## Simulations and application to real-life data

### Inference of gene order from distance data

The theory outlined above predicts that “well-behaved” gene clusters, i.e., those that (i) evolved by duplication of single genes only and (ii) did not experience rearrangements, should be Robinsonian. In other words, the shortest Hamiltonian path with respect to the genetic distances between its constituents should be co-linear with the genomic order. It is therefore of interest to study the length distribution of Hamiltonian paths. Associating a pseudo-energy $f(\pi )=\sum _{i=2}^n d(\pi _{i-1},\pi _i)$ with a path/permutation $\pi$ we may construct a probabilistic model where $Prob[\pi ] \propto \exp (-\beta f(\pi ))$ with an “inverse temperature” parameter $\beta$. Höner zu Siederdissen et al. (2014) and Höner zu Siederdissen et al. (2015) showed that this model is tractable by a variation of the well-known exponential-time dynamic programming approach to the Travelling Salesman Problem (Bellman 1962). In brief, the ensemble (*p*, *A*, *q*) of paths starting in *p*, ending in *q* and running through all elements of *A* is of the form $(p,A,q) = \bigcup _{u\in A} (p,A{\setminus }\{u\},u)\circ (u,q)$. Using a variant of algebraic dynamic programming on sets, this simple decomposition can be used to compute the posterior probabilities of adjacencies in the ensemble of Boltzmann-weighted paths as well as the posterior probabilities of vertices *p* and *q* to be endpoints of a Hamiltonian path. Further details on the method are discussed by Höner zu Siederdissen et al. (2014) and Höner zu Siederdissen et al. (2015). It is implemented in the Gene Cluster Evolution Determined Order software package Gene-CluEDO.^1^

**Fig. 6:**
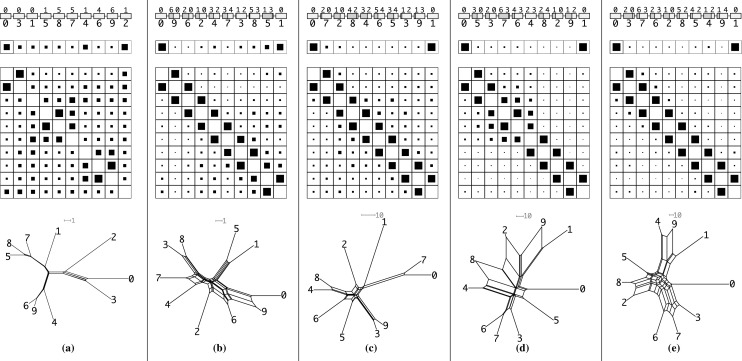
Examples of simulated gene clusters (see text for details). The mutation rate $\mu$ in the simulations are: in **a**, **b** 1%, in **c** 5%, in **d** 10%, in **e** 15%. Only in **a** local gene duplication events are employed as model while in **b**–**e** unequal crossing over events as proposed by Gehring’s model are used to create the cluster. Each column is a composite of four rows. In the first row the simulated cluster and its genes including their history is shown. Here, in the upper part the ancestral gene/genes are noted while on the bottom the simulated order is depicted. In the case of an unequal crossing over in Gehring’s model, the two parents are the number above the sequence block where the left number contributes the left (dark grey) part of the sequence and the right number the right part (light grey) of the sequence. In the second and third row the results of Gene-CluEDO are displayed. They are created with $\beta =0.01$. The size of the black box in a cell is proportional to the likelihood of this cell. The second row shows the probability that a sequence is on the edge of the cluster. The third row gives the probability that two sequences are adjacent to each other in the cluster. The last row then shows the network that is created with the NeighborNet (Bryant et al. 2004, 2007) algorithm. Note that the NeighborNets may scale differently indicated by a grey scale bar. Evolutionary distances are computed with emboss 6.6 (Rice et al. 2000) and are expressed as the number of substitutions per 100 characters

Since the genetic distance matrix is expected to have the Kalmanson properties the NeighborNet (Bryant et al. 2004, 2007) algorithm can be used as an alternative method to infer the expected gene order. The consistency theorem for NeighborNet (Bryant et al. 2004, 2007) in particular guarantees that the correct order will be obtained for ideal input data, i.e., input data that satisfies the Kalmanson condition. In practice, NeighborNet has turned out to be rather resilient to noise. Hence, it can be expected to produce good approximations to the gene order also for imperfect, noisy input data. Concurrence of Gene-CluEDO and NeighborNet can thus be used as support for the correctness of the reconstructed order, see Fig. 6.

### Simple simulation of gene cluster evolution

In order to test whether sequence evolution indeed approximates type R distances we generated artificial amino acid sequence data starting from a random initial sequence of length *N*. For the data reported here we use $N=1000$ and a uniform distribution of the 21 amino acids (including selenocystein). The initial sequence is copied identically; then both copies are independently mutated with a position-wise rate $\mu$ to generate the two initial parents. Subsequently, recombinant offspring are produced in an iterative fashion.

In each step, first a recombinant sequence *z* is produced from two adjacent parents *x* and *y* so that *z* is placed between *x* and *y*. To model unequal crossing over in Gehring’s model we randomly choose a breakpoint position *k* and produce *z* as a concatenation of *y*[1, *k*] and $x[k+1,n]$. In the first step, the initial sequence is simply copied. We also consider the case where the breakpoint is outside the “gene”, i.e., instead of producing a recombinant sequence *z* we use a copy of *x* or *y* with probability $\psi$. If $\psi =1$, we obtain the limit of tree-like evolution. The second part of each iteration step consists of independent mutations applied to all sequences. To this end, we replace with probability $\mu$ the amino acid in each sequence position by a randomly chosen alternative. The per site mutation rate $\mu$ must be chosen large enough to ensure a measurable divergence in each step. On the other hand, the sequence divergence should not saturate after *n* duplication-mutation steps, i.e., the expected total number of mutations per site should not substantially exceed 1. Thus $1/N \lesssim \mu \lesssim 1/n$.

Since we do not simulate insertions and deletions, the sequences are already properly aligned. In order to obtain an approximately additive distance matrix from the simulated sequences we use the Jukes–Cantor transformation (Jukes and Cantor 1969) to account for multiple mutations hitting the same site. We used emboss 6.6. (Rice et al. 2000) for this purpose. Fig. 6 shows data for a simulation with only local gene duplications in (a) and with unequal crossing over in Gehring’s model in each step in (b)–(e).

The gene order in the cluster and the reconstructed order in either the Gene-CluEDO or the circular order inferred using NeighborNet do not match for tree-like evolution. The reason is that in this case many orders, namely all outlines of any planar embedding of the tree, are equivalently perfect data. The simulated sequence data by construction contain stochastic noise that breaks this symmetry in a random manner. More precisely, distances empirically inferred from sequences will satisfy the equality in Eq. (3) only approximately. As a consequence, the tree edge belonging to the split *xy*|*uv* will be expanded to a narrow box as in Fig. 4. It is completely up to the noise, whether the second split is *xu*|*yv* or *xv*|*yu*, and thus, whether the circular order is *x*, *u*, *v*, *y* or *x*, *v*, *u*, *y*.

In contrast, both Gene-CluEDO and circular order reproduce the gene order in the cluster in the vast majority of simulations with unequal crossing over in Gehring’s model. The choice of the mutation rates $\mu$ makes little difference as long as the genetic distances between the sequences are not saturated.

An exception is Fig. 6c, where NeighborNet “misplaces” sequence 1. A detailed analysis of the data shows that both 3 and 9 are unequal crossing over products involving 1, however by chance the breakpoint was located so that only a tiny fraction of 1 was included in 3 and 9. The example thus contains an “almost tree-like” step, which does not retain sufficient ordering information.

### Analysis of gene clusters

#### Pairwise distances

In the following we illustrate the application of the theoretical results to the analysis of several gene clusters. To this end, we retrieved the amino acid sequence data of the annotated proteins from the NCBI database, constructed and—where necessary—manually curated sequence alignments, and used these to compute the matrices of pairwise genetic distances that are taken as input by both Gene-CluEDO and NeighborNet. Details on the data sources are compiled in the Online Supplement.

Multiple sequence alignments were computed with T-Coffee (Notredame et al. 2000). Since highly variable regions in the proteins mostly introduce noise into the alignment and the subsequent reconstruction of the phylogenetic network, we removed highly variable alignment columns using noisy (Dress et al. 2008). From the processed alignment we then computed the evolutionary distances interpreting gap characters as additional characters. The resulting raw distances are transformed into evolutionary distances using the Jukes–Cantor correction (Jukes and Cantor 1969). For the lancelet Hox cluster we obtained an extremely gap-rich alignment. We therefore constructed an alternative alignment using the block-based dialign approach (Al Ait et al. 2013), which identifies a chain of significant local alignments. We retained only the alignment blocks with a non-zero significance score.

**Fig. 7:**
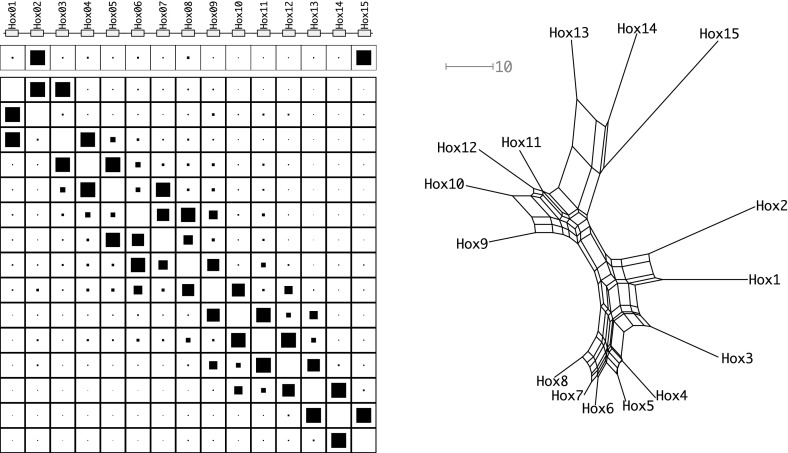
The Hox gene cluster of *B. lanceolatum*. How the pairwise distances are created is described in Sect. 4.3.1. The left site is a composite of three rows. The first row shows the cluster and the order on the genome. In the second and third row the results of Gene-CluEDO are displayed. They are created with $\beta =0.0025$. The size of the black box in a cell coincides with the likelihood of this cell. The second row shows the probability that a sequence is on the edge of the cluster. The third row gives the probability that two sequences are adjacent to each other in the cluster. The right side then shows the network that is created with the NeighborNet (Bryant et al. 2004, 2007) algorithm. The network scale is indicated by a grey scale, expressed as substitutions per 100 sites

#### Hox gene cluster

We already showed in previous work (Höner zu Siederdissen et al. 2015) that the Hamilton path method implemented in Gene-CluEDO can be applied to investigating the ancient evolution of Hox gene clusters. Cephalochordates harbour the largest known single Hox gene clusters, comprising 15 members (Pascual-Anaya et al. 2012). The Hox gene clusters are known to have expanded independently in the major deuterostome lineages (Pascual-Anaya et al. 2013) making them a particularly interesting model system for testing Gehring’s model. The results of this analysis are shown in Fig. 7. Overall, the amphioxus cluster behaves as expected. In line with the analysis of Hox clusters from the coelacanth (Höner zu Siederdissen et al. 2015), both Gene-CluEDO and NeighborNet reproduce the genomic arrangement. There are a few notable deviations, however: Both methods report a reversed ordering of HOX1 and HOX2. A blastp search, however, confirmed that the sequences of these two genes unambiguously belong to the HOX1 and HOX2 paralog groups that are present in all deuterostomes. We suspect that adaptive evolution of one of these genes may be responsible for the observed discrepancy. NeighborNet shows HOX11 and HOX12 in reverse order. However, the splits involved in establishing this ordering have very small weights, suggesting that this reversal is not significant.

We conclude, therefore, that the evolution of the HOX gene cluster most likely followed Gehring’s model. Another aspect supporting this conclusion is the placement of splits in the network created by NeighborNet. The genes are placed in a nearly perfect circle around the center of the network. Comparing its topology to the topologies of the clusters created by simulating Gehring’s model, we can see high similarity in the network structures (see Fig. 6). The source data can be seen in Supplemental Table 1.

**Fig. 8:**
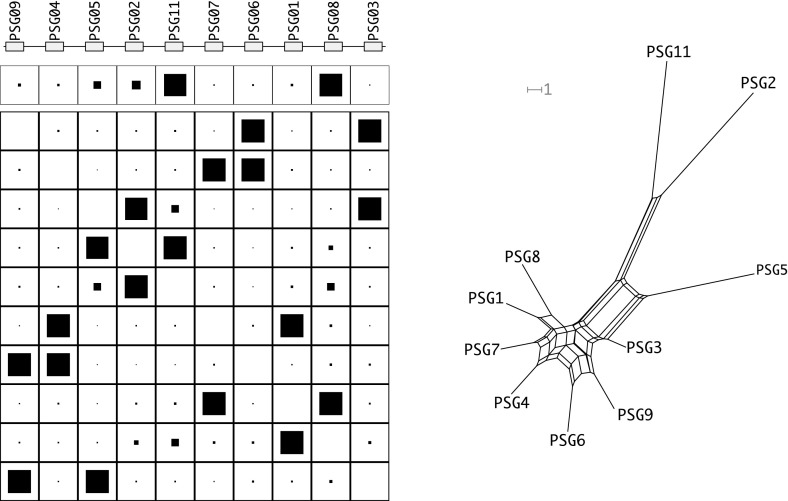
The PSG gene cluster of *Homo sapiens*. For additional legends see Fig. 7

#### PSG gene cluster

The pregnancy-specific glycoproteins (PSG) play an important role in the immune system during pregnancy (Chang et al. 2013). They form a well-defined subfamily of the Carcinoembryonales Antigen gene family, which in turn belongs to the immunoglobulin gene superfamily. The PSG family forms a cluster that has independently expanded in some mammalian classes, most prominently in rodents and primates. Here we analyzed the *human* PSG gene cluster, which contains ten PSG genes. Five CEACAM pseudogenes are interspersed in the cluster. The results of this analysis are shown in Fig. 8.

The data shows two remarkable properties. Consistent with evolutionarily recent duplications the PSG genes are very similar to each other. The second remarkable property is that the orders inferred with Gene-CluEDO and NeighborNet do not fit to the real genomic order. In fact only three (Gene-CluEDO) or four (NeighborNet) genes appear in the order of their genomic positions. The data are not consistent with the prediction from Gehring’s model.

Two aspects provide possible explanations. Zid and Drouin (2013) proposed that the PSG gene cluster in primates evolved under purifying selection for gene conversion. Chang et al. (2013) proposed that a high number of unequal crossing over events had occurred in primate evolution. A very large number of duplicates, however, may reduce the selection pressure on single gene copies such that gene loss is no longer lethal. This may lead to missing genes and to large differences in evolution rates of individual copies. The latter may account for a violation of the Robinson property, and thus deviations between the observed genomic gene order and the order inferred by Gene-CluEDO from the genetic distances. An observation that supports these explanations is that PSG11 and PSG2 stand out amongst the other genes as relatively diverse (see NeighborNet plot). Possibly genes that could close this gap were lost due to unequal crossing over. The source data can be seen in Supplemental Table 2.

**Fig. 9:**
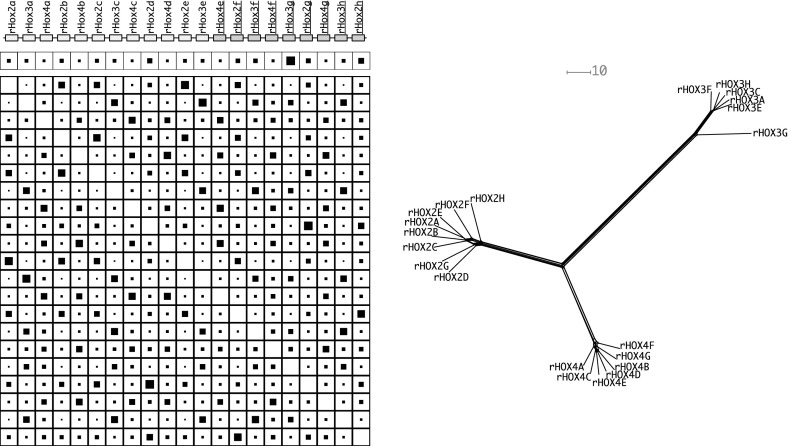
The $\alpha$-Rhox gene cluster of *Mus musculus*. Genes oriented in the opposite reading direction are indicated by darker boxes and underlined gene names. For additional legends see Fig. 7

#### $\alpha$-Rhox gene cluster

The Rhox genes (MacLean and Wilkinson 2010) are expressed during both embryogenesis and in adult reproductive tissues. In the mouse they are located in a single cluster on the X chromosome comprising 33 genes in three subclusters ($\alpha$, $\beta$ and, $\gamma$). The Rhox cluster is notable for its unusually rapid evolution. Here we included 23 well annotated genes of the $\alpha$-Rhox cluster, after removing the pseudogene rHox3d, the highly diverged rHox1 sequence, as well as rHox3b, for which no translation is reported in the NCBI database.

Figure 9 shows that the data set is divided into three groups. All rHox2 genes are in one group (left), all rHox3 genes form the second group (bottom) and all rHox4 genes build the third group (top right). These groups are clearly separated from each other. The $\alpha$-Rhox gene cluster clearly has not evolved conforming to Gehring’s model. As described e.g. by MacLean et al. (2006), the basic unit of tandem duplications is a block comprising an rHox2, rHox3, and rHox4 gene. Subsequent gene losses further restructured the cluster. In addition the cluster was subject to an inversion. Our analysis does not contradict this scenario. The source data can be seen in Supplemental Table 3.

**Fig. 10:**
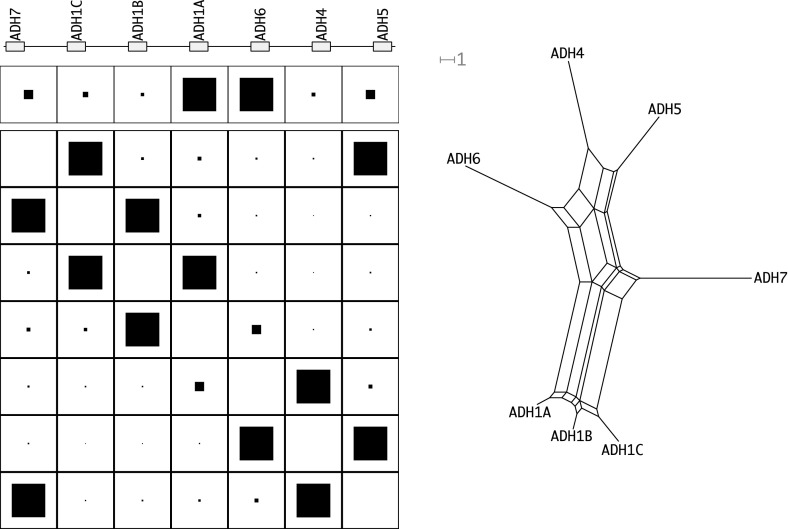
The ADH gene cluster of *Homo sapiens*. For additional legends see Fig. 7

#### ADH gene cluster

The alcohol dehydrogenases (ADH) family exists in a wide range of taxa, from bacteria to plants and humans (Oota et al. 2007). Their main function in animals is to break down alcohols that are otherwise toxic. Most members of this gene family appear in a well-studied gene cluster. The *Human* ADH gene cluster comprises seven genes, one each belonging to classes 2–5 as well as three paralogs of class 1 ADHs. Here, we find three elements in the cluster, which also cluster together regarding the results of Gene-CluEDO and NeighborNet, shown in Fig. 10.

As the genes are relatively similar to each other, genetic distances are small. The reconstructed cycle order inferred with both Gene-CluEDO and NeighborNet is the same as the genomic gene order. Gene-CluEDO identified ADH1A and ADH6 as the extreme ends in terms of genetic distance. These two genes are located adjacent to each other in the middle of the cluster. This may be an artefact of the small distances, since ADH5 and ADH7, for instance, have more or less the same distance to the split point inferred by Gene-CluEDO.

Our analysis thus suggests that the cluster evolved in line with Gehring’s model. The order is perfectly reconstructed. It has been argued by Oota et al. (2007) based on the observation that different exons of the genes resulted in different maximum parsimony trees that the ADH1 genes have not been subject to gene conversion (Oota et al. 2007). This observation is also consistent with the assumption of unequal crossing over within the gene as the mechanism underlying the duplications: in this scenario, duplicate genes are composed of two parts of two distinct genes, with different evolutionary history. Gene duplication following Gehring’s model therefore provides an explanation for the differences in exon-specific tree reconstructions as observed for ADH gene clusters. The source data can be seen in Supplemental Table 4.

## Conclusions

In this contribution we have investigated in some detail a model of gene cluster evolution that goes beyond identical tandem copies. Based on Walter Gehring’s ideas, we saw that unequal crossing over events produce genes that are hybrids of their adjacent genes. The distances between the members of a gene cluster therefore are not expected to be tree-like. Instead they form a distinctive subclass of circular decomposable (Kalmanson) distances, which we have termed here type R. As a consequence, the genomic gene order matches the circular order associated with the Kalmanson-type genetic distance matrix. The NeighborNet algorithm (Bryant et al. 2004), a commonly used tool for the inference of phylogenetic networks, readily infers this order. This provides a simple method to check whether a gene cluster evolves according to Gehring’s model or not. To better characterize type R distances, we showed that they are recognizable in polynomial time and that the sequence of unequal crossing over events can be inferred from a given type R distance matrix.

Additive tree metrics, which arise if the crossing over breakpoints are located between genes, are a special case of type R distances. In this case, the circular order is ambiguous since an arbitrary decision can be made at each interior vertex of the phylogenetic tree. More precisely, all planar embeddings of the phylogenetic tree yield a valid circular order.

The genetic distances of gene clusters evolving according to Gehring’s model of unequal crossing over within genes also satisfy the Robinson condition, at least as long as selective pressures and thus evolutionary rates on paralogous members are not too different. This implies that shortest Hamiltonian paths with respect to the genetic distance should be co-linear with the genomic order of genes. Numerical simulations show that this type of co-linearity can be used to distinguish clusters that evolve through unequal crossing over within genes from clusters where unequal crossing over occurs (mostly) between genes. The tree-like evolution in the latter case yields equivalent solutions of the shortest Hamiltonian path problem, again corresponding to arbitrary planar embeddings of the tree. Small amounts of noise in the data then typically yield optimal solutions that differ substantially from co-linearity with the genomic arrangement.

We tested these ideas using well-studied gene clusters as examples. The Hox cluster of the lancelet, for instance, essentially follows Gehring’s paradigm. This is also true to a certain extent for the ADH gene cluster. Other clusters, such as the cluster of rodent Rhox genes or the PSG immunoglobulins, however, show little or no indication of unequal crossing over within genes, and drastic deviations from co-linearity between gene orders inferred from genetic distances and their actual genomic arrangements.

The work presented here focused on the mathematical foundations and the demonstration that genetic distance matrices are informative about the mode of gene cluster evolution. Several open problems remain, in particular related to practical applications. The recognition algorithm Algorithm 2 requires an exact type R structure. Since the conditions for a metric to be type R involves equalities, an empirically determined distance matrix generically will not be type R due to noise. This raises the question how a best-fitting type R matrix can be identified, and how the deviation from a type R matrix should be quantified most appropriately. Together with the approximation of a type R matrix it would be useful to compute the most likely sequence of unequal crossing over events.

If a gene cluster evolves according to Gehring’s model with appreciable levels of gene conversion, we expect that values of *a* inferred from Eq. (9) are typically bounded away from 0 or 1. This implies that the type R matrix should be decidedly non-tree like. It remains a question for future work how the distribution of *a* values relates to well-established measures of deviations from tree-likeness, such as the parameters proposed by statistical geometry (Nieselt-Struwe 1997). Similarly, it remains an open problem how to properly quantify the deviation of a given metric from type R structure. As with circular decomposable metrics there does not seem to be an easy-to-compute measure. The split-prime part of the metric, i.e., the unique component that is not decomposable in split metrics (Bandelt and Dress 1992), might serve at least as a first approximation for this purpose. These issues, however, extend beyond the scope of the present contribution and will require a most systematic analysis of a larger number of gene clusters.

In this contribution we have considered only the special case that unequal crossing over is restricted to adjacent genes. This assumption does not cover all cases of biological interest, as the case of the Rhox cluster shows: there, the unit of duplication is a sequence of three genes. It will be interesting to see, whether unequal crossing over events that lead to the duplication of larger subclusters lead to similar mathematical structures, and whether such events could be inferred from a careful analysis of the genetic distance matrix.

### Electronic supplementary material

Below is the link to the electronic supplementary material.

Supplementary material 1 (pdf 107 KB)

## Predecessor relation of crossover events

Algorithm 3 utilizes the associative properties of gene identifiers that allow constant time mapping between pairs of genes and recombinant triples. If triples are derived from a linear type R matrix, they form a natural binary tree that is to be established by the algorithm, where each triple (*x*, *y* : *z*) can be a left or right successor to another triple on (*x*, *y*), accept a left successor on (*x*, *z*), or accept a right successor on (*z*, *y*).

Each triple is added in turn, checking for connections to already added triples using associative arrays (map) for each connection type. If a connected triple was already added, an open entry is found in the corresponding map, else a new entry will be added to the according inverse map. For instance, an added left successor needs to look at an open predecessor. If a single tree was created, the linear order of genes can be found by traversing the tree. If no linear order exists, multiple trees will be created, as necessary connectors are either never added to an open map or have been removed since entries in open maps are only used for a single connection.

## Data sources for the analysis gene clusters

Data sources relating to the gene clusters analyzed in this contribution as listed in the Electronic Supplemental Material. In addition, machine readable data, including alignments of all sequences used to compute genetic distances, are compiled for download at http://www.bioinf.uni-leipzig.de/publications/supplements/17-012.

## Footnotes

1 Sources: http://hackage.haskell.org/package/Gene-CluEDO binaries: https://github.com/choener/Gene-CluEDO/releases.
