# Supplementary material for: Expansion of gene clusters, circular orders, and the shortest Hamiltonian path problem
Source: J Math Biol. 2017 Dec 19;77(2):313–41. doi: 10.1007/s00285-017-1197-3 (PMC6060901; doi:10.1007/s00285-017-1197-3)
Supplement: Supplementary file 1 — Supplementary material 1 (pdf 107 KB) [file 285_2017_1197_MOESM1_ESM.pdf]

Noname manuscript No.
(will be inserted by the editor)

Expansion of Gene Clusters, Circular Orders, and the Shortest Hamiltonian Path Problem SUPPLEMENTAL MATERIAL

Sonja J. Prohaska · Sarah J. Berkemer · Fabian Gärtner · Thomas Gatter · Nancy Retzlaff · The Students of the Graphs and Biological Networks Lab 2017 · Christian Höner zu Siederdissen · Peter F. Stadler

the date of receipt and acceptance should be inserted later

Additional machine readable data, including alignments of all sequences used to compute genetic distances, are compiled for download at http://www.bioinf.uni-leipzig.de/publications/supplements/17-012.

S.J. Prohaska
Computational EvoDevo Group, Department of Computer Science & Interdisciplinary Center for Bioinformatics, Universität Leipzig, Härtelstraße 16-18, D-04107 Leipzig, Germany.
E-mail: sonja@bioinf.uni-leipzig.de

Sarah J. Berkemer and Nancy Retzlaff
Max Planck Institute for Mathematics in the Sciences, Inselstraße 22, D-04103 Leipzig, Germany

Fabian Gärtner
Competence Center for Scalable Data Services and Solutions Dresden/Leipzig and Bioinformatics Group, Department of Computer Science, Universität Leipzig, Härtelstraße 16-18, D-04107 Leipzig, Germany

C. Höner zu Siederdissen and Thomas Gatter
Bioinformatics Group, Department of Computer Science & Interdisciplinary Center for Bioinformatics, Universität Leipzig, Härtelstraße 16-18, D-04107 Leipzig, Germany;

P.F. Stadler
Bioinformatics Group, Department of Computer Science & Interdisciplinary Center for Bioinformatics, Universität Leipzig, Härtelstraße 16-18, D-04107 Leipzig, Germany; Max Planck Institute for Mathematics in the Sciences, Inselstraße 22, D-04103 Leipzig, Germany; RNomics Group, Fraunhofer Institute for Cell Therapy and Immunology, D-04103 Leipzig, Germany; Institute for Theoretical Chemistry, University of Vienna, Währingerstrasse 17, A-1090 Wien, Austria; Santa Fe Insitute, 1399 Hyde Park Rd., Santa Fe NM 87501, USA.
E-mail: studla@bioinf.uni-leipzig.de

Table 1 Overview of the sequence data in the Hox gene cluster of *Branchiostoma lanceolatum*. The Hox11 gene is only partially available and has been replaced here with the Hox11 gene of the closely related *Branchiostoma lanceolatum* Hox11 sequence, to which the available fragment is identical. Only the homodomains were used.

Full Name	Abbreviation	Accession	Database	URL
Hox1	Hox1	ACJ74382.1	GenBank	Hox1
Hox2	Hox2	ACJ74381.1	GenBank	Hox2
Hox3	Hox3	ACJ74380.1	GenBank	Hox3
Hox4	Hox4	ACJ74383.1	GenBank	Hox4
Hox5	Hox5	ACJ74385.1	GenBank	Hox5
Hox6	Hox6	ACJ74384.1	GenBank	Hox6
Hox7	Hox7	ACJ74388.1	GenBank	Hox7
Hox8	Hox8	ACJ74387.1	GenBank	Hox8
Hox9	Hox9	ACJ74386.1	GenBank	Hox9
Hox10	Hox10	ACJ74389.1	GenBank	Hox10
Hox11	*	AAF81909.1	GenBank	Hox11
Hox12	Hox12	ACJ74391.1	GenBank	Hox12
Hox13	Hox13	ACJ74390.1	GenBank	Hox13
Hox14	Hox14	ACJ74393.1	GenBank	Hox14
Hox15	Hox15	ACJ74394.1	GenBank	Hox15

Table 2 An overview of the sequence data in the PSG gene cluster.

Full Name	Abbreviation	Accession	Database	URL
Ppregnancy-specific beta-1-glycoprotein 1 isoform 2 precursor	PSG1	NP_001171754.1	NCBI Reference Sequence	PSG1
pregnancy-specific beta-1-glycoprotein 2 precursor	PSG2	NP_112536.2	NCBI Reference Sequence	PSG2
pregnancy-specific beta-1-glycoprotein 3 precursor	PSG3	NP_066296.2	NCBI Reference Sequence	PSG3
pregnancy-specific beta-1-glycoprotein 4 isoform 1 precursor	PSG4	NP_002771.2	NCBI Reference Sequence	PSG4
pregnancy-specific beta-1-glycoprotein 5 precursor	PSG5	NP_002772.3	NCBI Reference Sequence	PSG5
pregnancy-specific beta-1-glycoprotein 6 isoform a precursor	PSG6	NP_002773.1	NCBI Reference Sequence	PSG6
Putative pregnancy-specific beta-1-glycoprotein 7	PSG7	Q13046.2	UniProtKB/Swiss-Prot	PSG7
pregnancy-specific beta-1-glycoprotein 8 isoform a precursor	PSG8	NP_874366.1	NCBI Reference Sequence	PSG8
pregnancy-specific beta-1-glycoprotein 9 isoform 1 precursor	PSG9	NP_002775.3	NCBI Reference Sequence	PSG9
pregnancy-specific beta-1-glycoprotein 11 isoform 1 precursor	PSG11	NP_002776.3	NCBI Reference Sequence	PSG11

Table 3 An overview of the sequence data in the α-Rhox gene cluster.

Full Name	Abbreviation	Accession	Database	URL
reproductive homeobox 2A isoform 1	rHOX2A	NP_083479.1	NCBI Reference Sequence	rHOX2A
reproductive homeobox 2B	rHOX2B	NP_001092786.1	NCBI Reference Sequence	rHOX2B
reproductive homeobox 2C	rHOX2C	NP_001092788.1	NCBI Reference Sequence	rHOX2C
reproductive homeobox 2D	rHOX2D	NP_001075138.2	NCBI Reference Sequence	rHOX2D
reproductive homeobox 2E	rHOX2E	NP_001078817.1	NCBI Reference Sequence	rHOX2E
reproductive homeobox 2F	rHOX2F	NP_001078825.1	NCBI Reference Sequence	rHOX2F
reproductive homeobox 2G	rHOX2G	NP_001107625.1	NCBI Reference Sequence	rHOX2G
reproductive homeobox 2H	rHOX2H	NP_001093935.1	NCBI Reference Sequence	rHOX2H
reproductive homeobox 3A	rHOX3A	NP_918952.2	NCBI Reference Sequence	rHOX3A
reproductive homeobox 3C	rHOX3C	NP_001095927.3	NCBI Reference Sequence	rHOX3C
reproductive homeobox 3E	rHOX3E	NP_001171898.1	NCBI Reference Sequence	rHOX3E
reproductive homeobox 3F	rHOX3F	NP_001035178.3	NCBI Reference Sequence	rHOX3F
reproductive homeobox 3G	rHOX3G	NP_001138878.1	NCBI Reference Sequence	rHOX3G
reproductive homeobox 3H	rHOX3H	NP_001107629.1	NCBI Reference Sequence	rHOX3H
reproductive homeobox 4A	rHOX4A	NP_001034777.1	NCBI Reference Sequence	rHOX4A
reproductive homeobox 4B	rHOX4B	NP_067275.1	NCBI Reference Sequence	rHOX4B
reproductive homeobox 4C	rHOX4C	NP_001034778.1	NCBI Reference Sequence	rHOX4C
reproductive homeobox 4D	rHOX4D	NP_001034784.1	NCBI Reference Sequence	rHOX4D
reproductive homeobox 4E	rHOX4E	NP_957688.2	NCBI Reference Sequence	rHOX4E
reproductive homeobox 4F	rHOX4F	NP_001034785.1	NCBI Reference Sequence	rHOX4F
reproductive homeobox 4G	rHOX4G	NP_001034787.1	NCBI Reference Sequence	rHOX4G

Table 4 An overview of the sequence data in the ADH gene cluster.

Full Name	Abbreviation	Accession	Database	URL
alcohol dehydrogenase 7	ADH7	AAB38424.1	GenBank	ADH7
alcohol dehydrogenase 1C	ADH1C	NP_000660.1	NCBI Reference Sequence	ADH1C
alcohol dehydrogenase 1B isoform 1	ADH1B	NP_000659.2	NCBI Reference Sequence	ADH1B
alcohol dehydrogenase 1A	ADH1A	NP_000658.1	NCBI Reference Sequence	ADH1A
alcohol dehydrogenase 6 isoform 1	ADH6	NP_001095940.1	NCBI Reference Sequence	ADH6
alcohol dehydrogenase 4 isoform 1	ADH4	NP_001293101.1	NCBI Reference Sequence	ADH4
ADH5	ADH5	CAG38730.1	GenBank	ADH5
